# Detection of *Borrelia burgdorferi* (s.l.) in *Ixodes ricinus* ticks collected in Iceland

**DOI:** 10.1186/s13071-025-06809-9

**Published:** 2025-05-15

**Authors:** Matthias Alfredsson, Kayleigh Hansford, Daniel Carter, Heiða Sigurðardóttir, Þórunn Sóley Björnsdóttir, Hrólfur Smári Pétursson, Guðný Rut Pálsdóttir, Jolyon M. Medlock

**Affiliations:** 1 Natural Science Institute of Iceland, Urridaholtsstraeti 6–8, 212 Gardabaer, Iceland; 2 Medical Entomology Group, UK Health Security Agency, Porton Down, Salisbury, Wiltshire UK; 3 Genomics of Rare and Emerging Human Pathogens Department, UK Health Security Agency, Porton Down, Salisbury, Wiltshire UK; 4 Institute for Experimental Pathology at Keldur, University of Iceland, Keldnavegur 3, 112 Reykjavik, Iceland

**Keywords:** Tick-borne pathogens, *Borreliaburgdorferi* (s.l.), Lyme disease, Ticks, *Ixodesricinus*

## Abstract

**Background:**

*Ixodes ricinus* is the most common tick species throughout Europe; it can transmit various pathogens that can cause diseases in humans and animals. It is the principal vector of *Borrelia burgdorferi* sensu lato (s.l.) and tick-borne encephalitis virus (TBEV), and there is increasing concern about *I. ricinus*'s potential to transmit pathogens to humans and animals in Iceland. The aim of this research is to determine whether *I. ricinus* ticks collected in Iceland carry *B. burgdorferi* (s.l) and, in a limited number of samples, other pathogens to better understand the potential health risks that *I. ricinus* bites may pose to both humans and animals in Iceland.

**Methods:**

Birds were captured and examined for ticks at the South East Iceland Bird Observatory from 2018 to 2019. All ticks were screened for infection with *B. burgdorferi* (s.l.). Additionally, 133 ticks collected in Iceland prior to 2018, stored in the collection at the Natural Science Institute of Iceland, were screened for the presence of *B. burgdorferi* (s.l.), TBEV, *Coxiella burnetii*, *Francisella tularensis* and *Rickettsia* spp. Samples positive for *Borrelia* were sequenced by Genewiz Azenta, Germany, and BLAST (NCBI) analysis was performed on the obtained sequences.

**Results:**

A total of 1209 *I. ricinus* ticks collected in Iceland were screened for the presence of *B. burgdorferi* (s.l.); 133 ticks from a museum collection were additionally screened for other pathogens. *Borrelia burgdorferi* (s.l.) was detected in 9.9% of the tick samples (86/866). DNA sequencing from 28 positive samples revealed three genospecies. The most frequently detected was *Borrelia garinii* (82.1%), followed by *B. valaisiana* (14.3%) and *B. afzelii* (3.6%). TBEV, *C. burnetii*, *F. tularensis* and *Rickettsia* spp. were not detected in ticks from the collection.

**Conclusions:**

This research confirms the presence of *B. burgdorferi* (s.l.) in *I. ricinus* ticks collected in Iceland. Even though Lyme disease is not endemic and *I. ricinus* ticks are not considered established, the risk of exposure remains. Further research on *B. burgdorferi* (s.l.) and other pathogens these ticks may carry is essential along with raising public awareness and fostering collaboration between experts to reduce the risk of tick-borne diseases in Iceland.

**Graphical Abstract:**

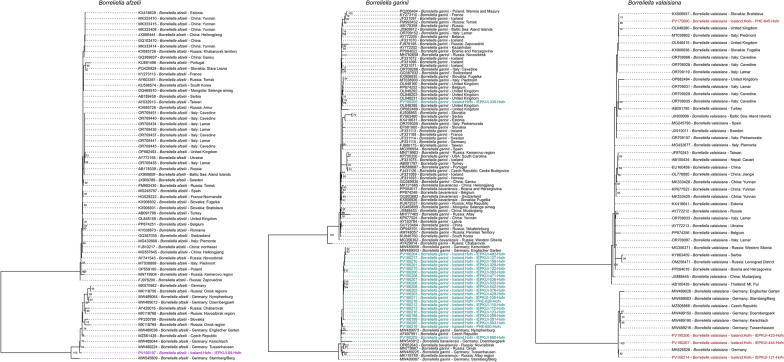

**Supplementary Information:**

The online version contains supplementary material available at 10.1186/s13071-025-06809-9.

## Background

Ticks (Acari: Ixodida) are the primary vectors for several arthropod-borne pathogens in Europe [1–3]. In Europe, ticks have increased in numbers, expanded their northern distribution and are being found in higher altitude than before [4–9]. These changes in tick distribution pose new threats to areas that were previously free of tick-borne diseases [6–11].

*Ixodes ricinus* (Linnaeus, 1758) is the most common tick species throughout Europe [12]. It is a three-host tick that can feed on most terrestrial vertebrates sharing its habitat [13, 14]. The immature stages can be found on hosts of all sizes but adults are mostly found on larger hosts [5, 14]. *Ixodes ricinus* has the ability to transmit bacterial, viral, and protozoan pathogens that can cause diseases to humans and animals [15, 16].

*Borrelia burgdorferi* sensu lato (s.l.) is a complex consisting of at least 20 bacterial genospecies, nine of which occur in Europe [17, 18]. These genospecies are *Borrelia afzelii*, *B. bavariensis*, *B. bissettiae*, *B. burgdorferi* sensu stricto (s.s.), *B. garinii*, *B. lusitaniae*, *B. spielmanii*, *B. turdi* and *B. valaisiana* but new genospecies are discovered each decade. Within the *B. burgdorferi* (s.l.) complex, *B. afzelii*, *B. burgdorferi* (s.s.) and *B. garinii* are the most recognised agents of Lyme borreliosis [19, 20], which is the most prevalent tick-borne disease in Europe and North America [16, 21, 22]. Other genospecies are considered to have low pathogenicity and have been associated with few cases [18, 23, 24]. In Europe, *I. ricinus* is known to be the main vector for *B. burgdorferi* (s.l.), but this is *Ixodes scapularis* in North America and *I. persulcatus* in Asia and some parts of Europe [25–27]. *Ixodes ricinus* is also the principal vector for tick-borne encephalitis virus (TBEV). Various species of *Rickettsia* bacteria are also transmitted by hard ticks in Europe such as *Rickettsia helvetica*, *R. monacensis* and *R. slovaca*, among others, which are associated with human diseases [28–30].

*Ixodes ricinus* spends most of its lifecycle off the host, but to develop, reproduce and transmit *B. burgdorferi* (s.l.) it needs blood meals from vertebrate hosts [14]. Many vertebrate species, particularly small mammals and ground-foraging birds, serve as reservoir hosts for *B. burgdorferi* (s.l.) in Europe [28, 31, 32]. *Borrelia garinii*, *B. valaisiana* and *B.* turdi are associated with birds [33, 34], while *B. afzelii*, *B. bavariensis*, *B. spielmanii* and *B. burgdorferi* (s.s) are known to parasitise small- and medium-sized mammals [14, 35–37]. *Borrelia lusitaniae* is known to use reptiles, but also birds and small mammals, as reservoirs [38].

Ticks are commonly found on migratory birds and their continental dispersal by birds is well known [39–41]. It is the probably route for ticks to extend their distribution to areas that are less accessible to terrestrial mammals such as islands [42–47]. Passerine birds have been shown to be important pathogen reservoirs and carriers of infected ticks to less accessible areas [43–47]. Nevertheless, the maintenance of *B. burgdorferi* (s.l.) depends on small- and medium-sized mammals, birds or reptiles to acquire the infection from nymphs and transmit to larvae [14]. Host infectivity to susceptible ticks and host and tick population abundance are important factors in the transmission of *B. burgdorferi* (s.l.), and these factors vary across locations [14, 48, 49]. In areas with few ticks, the number of nymphal bites on small mammals might be too low to ensure maintenance of the transmission cycle.

The risk of acquiring tick-borne diseases such as Lyme borreliosis is often associated with deciduous and mixed woodlands where, unlike in Iceland, *I. ricinus* can be found in high numbers in other parts of Europe [50–53].

The aim of this research is to determine whether *I. ricinus* ticks collected in Iceland carry *B. burgdorferi* (s.l.) and, in a limited number of samples, other pathogens. To our knowledge, this is the first occasion where *I. ricinus* ticks from Iceland have been tested for *B. burgdorferi* (s.l). The findings are a vital step towards understanding the potential health risks that an *I. ricinus* bite may pose to both humans and animals in Iceland.

## Methods

### Bird trapping and collection of ticks

Ticks have been collected using a passive surveillance scheme in Iceland since 1976 and by active surveillance since 2015 when regular surveys, using the tick flagging method [54], were implemented. Additionally, in 2016–2017 bird ringers at the South East Iceland Bird Observatory were asked to check whether birds, especially migratory birds, had ticks on them and collect them. Through the passive and active surveillance schemes, a total of 133 ticks (larvae and nymphs) were collected and stored in a museum collection at the Natural Science Institute of Iceland prior to 2018. Of those 133 ticks, 20 were older museum specimens from the passive surveillance scheme, 17 specimens were collected during vegetation surveys, and 96 were bird-derived. All ticks were stored in tubes filled with 80% ethanol. These ticks were brought to UK Health Security Agency (UKHSA) and tested for *B. burgdorferi* (s.l.), TBEV, *C. burnetii*, *F. tularensis* and *Rickettsia* spp.

In 2018—2019, surveillance on migratory birds was established at South East Iceland Bird Observatory at Hofn, southeastern Iceland, using bird nets and a Heligoland trap to trap the birds. All captured birds were examined for tick infestations. Ticks were carefully removed with tweezers, preserved in 80% ethanol and identified using the keys of Hillyard [54] and Arthur [55]. Ticks collected from the Bird Observatory were screened for infection with *B. burgdorferi* (s.l.) at the Institute for Experimental Pathology at Keldur, University of Iceland (IEPKUI).

### Prevalence of ticks and mean ticks per infested bird

The prevalence of tick infestations on birds was calculated as the proportion of infested birds out of the total number of examined birds, expressed as a percentage:

$$Prevalence \left(\%\right)=\left(\frac{Number\, of\, infested\, birds}{Total\, number\, of\, examined\, birds}\right)x100$$

The mean number of ticks per infested bird was calculated by dividing the total number of ticks from all infested birds by the number of infested birds.

$$Mean\, ticks\, per\, infested\, bird=\frac{Total\, number\, of\, ticks\, from\, infested\, birds}{Number\, of\, infested\, birds}$$

### Homogenisation of ticks and extraction of nucleic acids

Nymphs were identified and then placed into individual Precellys homogenisation tubes (#MK28-R, Bertin Technologies) containing 250 μl of phosphate-buffered saline. Larvae were pooled eight individuals per tube.

Tubes containing ticks were placed into a Precellys 24 homogeniser (Bertin Technologies) and homogenised using three replicates of 30-s, 4000 rpm shakes. Nucleic acids were extracted from the homogenate using a QIAamp DNA Mini Kit (#51306, Qiagen Ltd.) according to the following protocol. An additional 250 μl of phosphate buffered saline was added to tubes for ticks that were not fully homogenised and homogenisation was repeated.

Wash buffers provided in the QIAamp DNA Mini Kit were reconstituted using 100% absolute ethanol (#459836, Merck) to the appropriate volumes. Nucleic acids were then extracted following the manufacturer's recommended protocol using 100 μl of tick homogenate and eluted in 100 μl AE buffer.

### PCR of tick extracts

Nucleic acid extracts were tested against a panel of tick-borne pathogens by adding 5 μl nucleic acid extract to 15 μl prepared qRT-PCR Mastermix. qRT-PCR reactions were prepared by using the QIAgility (Qiagen) automated PCR liquid handling instrument with qRT-PCR master mixes prepared following the reaction volumes described in Additional file 1: Tables S1–S5. Pathogens, targets and the associated oligonucleotide sequences used for the PCR screen are described in Additional file 1: Tables S1–S5.

### Tick-borne pathogen PCR panel oligonucleotide sequences

#### Borrelia, coxiella and francisella qPCR oligonucleotides

Cycling conditions using the QuantStudio 6 Flex (Applied Biosystems) were as described in Additional file 1: Table S6. Details on the primers and probes used to detect *Borrelia* spp., *C. burnetii* and *F. tularensis* are provided in Additional file 1: Table S7.

### Rickettsia and TBEV qPCR oligonucleotides

Cycling conditions performed using the LightCycler 480 (Roche) were as described in Additional file 1: Table S8. Details on the primers and probes used to detect *Rickettsia* spp. and TBEV are provided in Additional file 1: Table S7.

### *Borrelia* 5S–23S intergenic spacer PCR genotyping assay oligonucleotides

Cycling conditions performed using the 2720 thermocycler (Applied Biosystem) were as described in Additional file 1: Table S9. Details on the primers and probes used to detect *Borrelia* 5S–23S intergenic spacer are provided in Additional file 1: Table S7.

### Borrelia sequencing/genotyping

All samples positive for *Borrelia* by qPCR were genotyped using the *Borrelia* 5S-23S intergenic spacer PCR with the oligonucleotides described in Additional file 1: Tables S5, S9. Reactions were carried out with the following reaction volumes described in Additional file 1: Table S5 and cycling conditions performed in a 2720 thermocycler (Applied Biosystems) using the conditions described in Additional file 1: Table S:9. The PCR products were analysed by electrophoresis in a QIAxcel Advanced System and visualised using the QIAxcel Gel Software. PCR products showing a clear 420-bp band were purified using the Monarch PCR & DNA Cleanup Kit (New England Biolabs Inc.) according to the manufacturer’s protocol. Sanger sequencing was done by Genewiz Azenta, Germany, and the raw data were edited and analysed using the Sequencher^®^ version 5.4.6 DNA sequence analysis software (Gene Codes Corp., Ann Arbor, MI). Finally, a BLAST (NCBI) analysis was performed on the obtained sequences to compare them with reference sequences for species identification.

### Phylogenetic analysis

Icelandic *Borrelia* 5S-23S rRNA intergenic spacer sequences and range of relevant reference sequences were aligned using MAFFT [64] for each different genotype. Maximum likelihood phylogenetic trees were then constructed using IQ-TREE [65] using MFP, HKY, G4 and I model with a bootstrap setting of 1000. Phylogenetic trees were then visualised and edited using R Studio [66] and the ggtree package [67].

## Results

A total of 1209 *I. ricinus* ticks (512 larvae and 697 nymphs), collected in Iceland from 1999 to 2019, were screened for the presence of *B. burgdorferi* (s.l.) at the laboratories of UKHSA (*n* = 133) and IEPKUI (*n* = 1076). At UKHSA, ticks were also screened for the presence of TBEV, *C. burnetii*, *F. tularensis* and *Rickettsia* spp. Ticks screened at UKHSA comprosed all available *I. ricinus* larvae and nymphs in Iceland at the time, 20 nymphs from the museum collection at the Natural Science Institute of Iceland (NSII), acquired using a passive surveillance scheme, 17 nymphs acquired through vegetation surveys and 96 bird-derived ticks (32 larvae and 64 nymphs). At IEPKUI all ticks were bird-derived and had been collected at the South East Iceland Bird Observatory from 2018 to 2019. A total of 3429 birds of 26 species were captured during that time and every bird checked for ticks. These 1076 *I. ricinus* ticks (480 larvae and 596 nymphs) were collected from 208 birds. All infested birds were redwings (*Turdus iliacus*) except for one blackbird (*Turdus merula*) and one willow warbler (*Phylloscopus trochilus*) with tick infestation prevalence of 6.1% of the total birds examined. Tick infestation prevalence for redwings was 10.8% with a mean of 5.2 ticks per infested bird. The highest number of ticks was found on a redwing, 86 ticks (17 nymphs and 69 larvae) (Table 1).

**Table 1:** Tick infestation of birds captured and examined at the South East Iceland Bird Observatory 2018–2019

Bird species	No. of birds examined	No. of birds infested	Infested/examined (%)	No. of ticks (nymphs/larvae)	Mean ticks per infested bird
*Turdus iliacus* (Redwing)°	1908	206	10.8	1061 (584/477)	5.2
*Anthus pratensis* (Meadow pipit)	700				
*Acanthis flammea* (Common redpoll)*	298				
*Motacilla alba* (White wagtail)	285				
*Oenanthe oenanthe* (Northern wheater)	64				
*Troglodytes troglodytes* (Eurasian wren)*	52				
*Gallinago gallinago* (Common snipe)	30				
*Sylvia atricapilla* (Eurasian blackcap)	19				
*Turdus merula* (Common blackbird)°	16	1	6.3	14 (12/2)	14.0
*Fringilla montifringilla* (Brambling)	11				
*Turdus philomelos* (Song thrust)	11				
*Vireo olivaceus* (Red-eyed vireo)	9				
*Phylloscopus trochilus* (Willow warbler)	6	1	16.7	1 (0/1)	1.0
*Erithacus rubecula* (European robin)	4				
*Phylloscopus inornatus* (Yellow-browed warbler)	3				
*Falco columbarius* (Merlin)	3				
*Fringilla coelebs* (Eurasian chaffinch)	1				
*Tadorna tadorna* (Common shelduck)*	1				
*Phylloscopus collybita* (Common chiffchaff)	1				
*Loxia curvirostra* (Red crossbill)°	1				
*Hirundo rustica* (Barn swallow)	1				
*Turdus viscivorus* (Mistle thrust)	1				
*Catharus ustulatus* (Swainson´s thrust)	1				
*Sylvia curruca* (Lesser whitethroat)	1				
*Anas platyrhynchos* (Mallard)*	1				
*Loxia leucoptera* (Two-barred crossbill)	1				
Total	**3429**	**208**	**6.1**	**1076 (480/596)**	**5.2**

^*^Resident birds, °both resident and migratory birds

All nymphs were individually screened for pathogens, but the larvae were pooled together, eight per tube at UKHSA and one to four per tube at IEPKUI (only larvae from individual birds were pooled together), for a total of 866 samples (697 nymphs and 169 pooled larvae samples). *Coxiella burnetii*, *F. tularensis*, *Rickettsia* spp. and TBEV were not detected in the samples tested at UKHSA (*n* = 133). *Borrelia burgdorferi* (s.l.) DNA was detected in 9.9% (86/866) of all the *I. ricinus* samples, 10.2% (71/697) of all nymphs and 8.9% of the larvae samples (Table 2). The larvae were all blood-fed and probably acquired the *Borrelia* from the bird host. Of the infected ticks, 93 were collected from redwings and 14 from a blackbird, one questing tick and one from the museum collection. It was possible to sequence *B. burgdorferi* (s.l.) DNA from 28 positive samples, 25 of them collected from a redwing and three from a blackbird. DNA sequencing revealed three genospecies: The most frequenctly detected genospecies was *B. garinii* (82.1%) followed by: *B. valaisiana* (14.3%) and *B. afzelii* (3.6%). Isolates from this study, the *Borrelia* genotype detected and their associated GenBank accession numbers are included in Additional file 1: Table S10.

**Table 2:** Total number of tested tick samples

	No. of samples	Bird derived from	Questing	Museum collection	No. of positive samples for *Borrelia* spp. by qPCR (%)	No. of samples positive for *Borrelia garinii* (%)	No. of samples positive for *Borrelia valaisiana* (%)	No. of samples positive for *Borrelia afzelii* (%)
Larvae	169	169	0	0	15 (8.9)	7 (25.0)		
Nymphs	697	660	17	20	71 (10.2)	16 (76.2)	4 (14.3)	1 (3.6)
Total	**866**	**829**	**17**	**20**	**86 (9.9)**	**23 (82.1)**	**4 (14.3)**	**1 (3.6)**

Larvae samples were pooled (1–8) per tube, while nymphs were tested individually. The number of positive samples (%) for *Borrelia* spp. was determined by qPCR

**Fig. 1:**
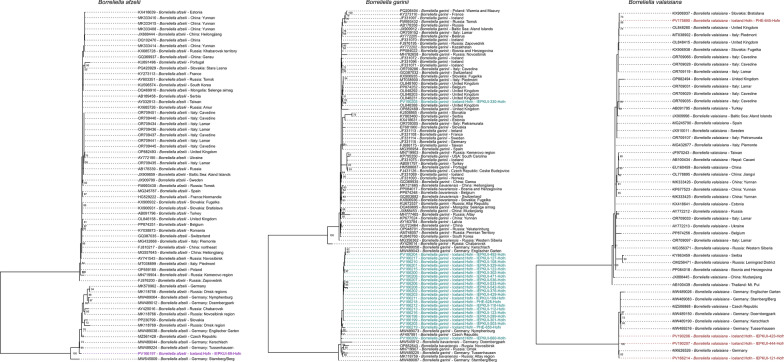
Maximum likelihood phylogeny of the Icelandic nucleotide sequences for *Borrelia afzelii*, *B. garinii* and *B. valaisiana* strains that cluster within each representative genotype. The phylogeny was constructed using IQ-TREE and is midpoint rooted, with bootstrap values > 50 displayed at each relevant node. Sequences generated during this study are highlighted in purple for *B. afzelii*, cyan for *B. garinii* and red for *B. valaisiana*. The Icelandic *B. afzelii* sequence clusters with strains sequenced in central and eastern Europe whilst *B. garinii* and *B. valaisiana* sequences cluster with either central and eastern Europe or the UK

Phylogenetic analysis of the 5S-23S rRNA intergenic spacer sequences generated confirms the classification of each relevant *Borrelia* genospecies initially identified by BLAST analysis. Phylogenetic trees for the three *Borrelia* genotypes identified (*B. afzelii*, *B. garinii* and *B. valaisiana*) are included in Fig. 1. For the single *B. afzelii* sequence generated, the Icelandic strain clustered closely with strains sequenced from Germany, Czech Republic, Russia and Slovakia. For *B. garinii*, most Icelandic sequences again clustered with strains sequenced from Germany, Russia and Czech Republic. A single sequence however clustered with sequences generated from the UK. For *B. valaisiana*, three Icelandic sequences clustered with strains sequenced from Germany and Czech Republic. A single Icelandic sequence clustered with sequences from UK, Slovakia and Italy.

## Discussion

The objective of this study was to investigate whether *I. ricinus* ticks collected in Iceland could harbour pathogens such as *B. burgdorferi* (s.l.). *Ixodes ricinus* is the principal vector of several tick-borne diseases, including Lyme borreliosis, and there is increasing concern about its potential to transmit these pathogens to humans and animals in Iceland. Lyme borreliosis is not considered to be endemic, no cases of Icelandic origin have been published [68], and according to the Icelandic Director of Health, there have been no recorded domestic cases of TBE. It is well established that ticks transported by migratory birds from other countries may carry pathogens. This had not been confirmed for ticks collected within Iceland until now. Our findings revealed that *B. burgdorferi* (s.l.) was detected in 9.9% of the 866 *I. ricinus* samples tested. DNA sequencing of 28 positive samples identified three *Borrelia* genospecies: *Borrelia garinii*, *B. valaisiana* and *B. afzelii*. The most frequently detected genospecies were *B. garinii* (82.1%) and *B. valaisiana* (14.3%), both of which are known to have passerine birds as reservoir hosts [43–47], which aligns with other studies that have focused on bird-derived ticks from neighbouring countries [69–71]. This result was expected given that most ticks were collected from migratory birds. Interestingly, one nymph was infected with *B. afzelii* (3.6%), a genospecies typically associated with rodents, suggesting that this nymph may have fed on rodents during the larval stage before being transported to Iceland.

Iceland's passive surveillance scheme has recorded over 400 *I. ricinus* ticks to date (NSII unpublished data). However, our study collected and identified 1076 ticks from 208 birds captured over 2 years (2018–2019) at the South East Iceland Bird Observatory station. In this study, 98% of ticks were collected from redwings, underscoring the potential role of this bird species in introducing ticks to Iceland. No host-seeking larvae have been found in Iceland, and larvae have not been detected on either rodents or resident birds. Several factors, particularly the lack of suitable hosts, likely inhibit tick establishment [42]. While it is possible that a small local population exists, none has been identified despite considerable efforts.

At UKHSA, we were able to test 133 tick samples from Iceland for a range of pathogens, comprising all available larvae and nymphs at the time. The following year, we realised that sampling ticks from birds during bird ringing was an especially effective method of collecting large numbers of ticks. This led to the decision to investigate the possibility of conducting testing within Iceland, utilising the expertise gained from UKHSA. Unfortunately, lack of funding limited the scope of testing, so we focused exclusively on *B. burgdorferi* (s.l.). Although it would have been ideal to test for a broader range of pathogens, this remains a future possibility should additional funding become available. One limitation in our study was the practice of storing all ticks collected from the same bird in a single tube. Ideally, each tick should have been stored separately to prevent cross-contamination. For example, we collected 14 ticks from a single blackbird, three of which were damaged during removal. All 14 ticks tested positive for *B. burgdorferi* (s.l.), and DNA sequencing of two ticks revealed the *B. garinii* genotype. Given that this genotype is primarily associated with birds, it is possible that all of the ticks were infected through their blood meal or co-feeding. However, there remains a possibility that the damaged ticks contaminated the others, which highlights the need for improved sample handling. It should also be mentioned that it is not the best method to store ticks in 80% ethanol since it could potentially reduce our chances of finding viruses like TBEV. In future studies we recommend placing ticks straight into buffers for RNA preservation to stabilise RNA immediately and protect it from degradation. Samples should then be transferred to − 20 °C or − 80 °C long-term storage.

Phylogenetic analysis indicated that most *Borrelia* genotypes sequenced during this study clustered with sequences from central and eastern European regions. This may indicate a key bird migratory route involved in the regular migratory movements of birds as well as the ticks and pathogens they carry across the Western Palearctic region [72]. The clustering of a small number of *B. garinii* and *B. valaisiana* sequences with sequences identified in the UK may also highlight a separate bird migratory route involved in the movement of *Borrelia*-infected ticks between Iceland and the British Isles. Interestingly, Icelandic *B. garinii* sequences from this study do not cluster with previously sequenced *B. garinii* detected in *Ixodes uriae* ticks collected in coastal regions of Iceland in 2011. This indicates that certain seabirds may not act as bridging hosts allowing the movement of *Borrelia* from imported *I. uriae* to native *I. ricinus* populations on the islands off the coast of Iceland. The detection of *Borrelia* in native *I. ricinus*, therefore, may be primarily linked to the movements of birds such as passerines.

There are some key limitations however to the phylogenetic analysis performed during this study and results are indicative only. Phylogeny inferred using the 5S-23S rRNA intergenic spacer region alone may be insufficient because of the relatively short fragment lengths amplified leading to limited variation between strains. There is also a potential bias due to the low availability of sequences, which are often limited to a small number of countries and regions which could make identification of specific routes of importation unclear. Future projects should focus on collection and testing of ticks from underreported regions for *Borrelia* and other tick-borne pathogens. This will provide more clarity about geographic links between different *Borrelia*-endemic regions and assist with identifying potential routes of movement of ticks and pathogens. Obtaining higher resolution sequence data using either Multi-locus Sequence Typing (MLST) or whole-genome sequencing will also provide greater confidence in these comparisons [73].

Collaboration among ornithologists, entomologists, microbiologists and healthcare professionals will be crucial in addressing this emerging issue. In addition, researching the effects of temperature, humidity and other environmental factors on tick survival will provide insight into the potential long-term risks posed by tick-borne diseases in Iceland. Public awareness campaigns should be initiated to educate local communities and healthcare workers about the risks associated with *I. ricinus* ticks. Whilst the primary aim of this study was to detect *B. burgdorferi* (s.l.), it is equally important to consider the possibility of other pathogens being carried by these ticks. Future research should focus on investigating the diversity and prevalence of these pathogens, providing a more comprehensive understanding of the health risks associated with *I. ricinus* ticks in Iceland.

## Conclusions

This research confirms the presence of *B. burgdorferi* (s.l.) in *I. ricinus* ticks collected in Iceland, raising concerns about the potential transmission of tick-borne diseases to humans and animals in Iceland. Although there is no evidence of an established local tick population, ongoing surveillance remains vital. These findings emphasise the need for sustained monitoring of ticks, their wildlife hosts and environmental factors. Further research on other pathogens these ticks may carry is essential, and raising public awareness, along with fostering collaboration between experts, will be crucial for managing and reducing the risk of tick-borne diseases in Iceland.

## Supplementary Information

Additional file 1. Table S1. Reaction volumes for the *Borrelia* spp. qPCR assay. Table S2. Reaction volumes for the *Coxiella burnetii* and *Francisella tularensis*. qPCR assays. Table S3. Reaction volumes for the *Rickettsia* spp. qPCR assay. Table S4. Reaction volumes for the TBEV. qPCR assay. Table S5. Reaction volumes for the *Borrelia *5S-23S rRNA intergenic spacer assay. Table S6. Cycling conditions optimised locally and based on the conditions described in the TagMan Fast Universal PCR Mastermix Manual. Table S7. Details on the primers and probes used to detect target microorganisms. Table S8. Cycling conditions optimised locally and based on the conditions described by Drosten et al., 2002. PCR Kit: Superscript III Platinum One-step qRT-PCR kit. Table S9. Cycling conditions optimised locally and based on the conditions described in the Tag 2X Mastermix Manual. Table S10. *Borrelia *genotypes detected by sequencing the 5S-23S rRNA intergenic spacer.

## Data Availability

No datasets were generated or analysed during the current study.

## Abbreviations

NSII: Natural Science Institute of Iceland
IEPKUI: Institute for Experimental Pathology at Keldur, University of Iceland
UKHSA: UK Health Security Agency
TBEV: Tick-borne encephalitis virus
