# Supplementary material for: Detection of Borrelia burgdorferi (s.l.) in Ixodes ricinus ticks collected in Iceland
Source: Parasit Vectors. 2025 May 15;18:176. doi: 10.1186/s13071-025-06809-9 (PMC12082893; doi:10.1186/s13071-025-06809-9)
Supplement: Supplementary file 1 — Additional file 1. Table S1. Reaction volumes for the Borrelia spp. qPCR assay. Table S2. Reaction volumes for the Coxiella burnetii and Francisella tularensis. qPCR assays. Table S3. Reaction volumes for the Rickettsia spp. qPCR assay. Table S4. Reaction volumes for the TBEV. qPCR assay. Table S5. Reaction volumes for the Borrelia 5S-23S rRNA intergenic spacer assay. Table S6. Cycling conditions optimised locally and based on the conditions described in the TagMan Fast Universal PCR Mastermix Manual. Table S7. Details on the primers and probes used to detect target microorganisms. Table S8. Cycling conditions optimised locally and based on the conditions described by Drosten et al., 2002. PCR Kit: Superscript III Platinum One-step qRT-PCR kit. Table S9. Cycling conditions optimised locally and based on the conditions described in the Tag 2X Mastermix Manual. Table S10. Borrelia genotypes detected by sequencing the 5S-23S rRNA intergenic spacer. [file 13071_2025_6809_MOESM1_ESM.docx]

**Additional file 1:**

Table S1 Reaction volumes for the Borrelia spp. qPCR assay.

| **Reagent** | **Manufacturer** | **Volume per reaction (μl)** | **Final concentration** |
| --- | --- | --- | --- |
| Nuclease-free water | Invitrogen (10320995) | 4.59 | N/A |
| TaqMan Fast Universal mastermix | Applied Biosystems (4352042) | 10 | 1X |
| Borrelia forward primer (100μM) | Eurofins | 0.18 | 900nM |
| Borrelia reverse primer (100μM) | Eurofins | 0.18 | 900nM |
| Borrelia probe (100μM) | Eurofins | 0.05 | 250nM |

Table S2 Reaction volumes for the C. burnetii and F. tularensis. qPCR assays.

| **Reagent** | **Manufacturer** | **Volume per reaction (μl)** | **Final concentration** |
| --- | --- | --- | --- |
| Nuclease-free water | Invitrogen (10320995) | 4.71 | N/A |
| TaqMan Fast Universal mastermix | Applied Biosystems (4352042) | 10 | 1X |
| C. burnetii / F. tularensis forward primer (100μM) | Eurofins | 0.18 | 900nM |
| C. burnetii / F. tularensis reverse primer (100μM) | Eurofins | 0.06 | 300nM |
| C. burnetii / F. tularensis probe (100μM) | Eurofins | 0.05 | 250nM |

Table S3 Reaction volumes for the Rickettsia spp. qPCR assay.

| **Reagent** | **Manufacturer** | **Volume per reaction (μl)** | **Final concentration** |
| --- | --- | --- | --- |
| Nuclease-free water | Invitrogen (10320995) | 3.81 | N/A |
| 2X Reaction mix | Invitrogen (11732-020) | 10 | 1X |
| Superscript III RT/Platinum Taq | Invitrogen (11732-020) | 0.8 | 2U |
| Rickettsia forward primer (100μM) | Eurofins | 0.18 | 900nM |
| Rickettsia reverse primer (100μM) | Eurofins | 0.09 | 450nM |
| Rickettsia probe (100μM) | Eurofins | 0.12 | 600nM |

Table S4 Reaction volumes for the TBEV qPCR assay.

| **Reagent** | **Manufacturer** | **Volume per reaction (μl)** | **Final concentration** |
| --- | --- | --- | --- |
| Nuclease-free water | Invitrogen (10320995) | 2.27 | N/A |
| 2X Reaction mix | Invitrogen (11732-020) | 10 | 1X |
| MgSO_4_ (50mM)  Superscript III RT/Platinum Taq | Invitrogen (11732-020)  Invitrogen (11732-020) | 1.6  0.8 | 4mM  2U |
| TBEV forward primer (100μM) | Eurofins | 0.18 | 900nM |
| TBEV reverse primer (100μM) | Eurofins | 0.1 | 450nM |
| TBEV probe (100μM) | Eurofins | 0.05 | 600nM |

Table S5 Reaction volumes for the Borrelia 5S-23S rRNA intergenic spacer assay.

| **Reagent** | **Manufacturer** | **Volume per reaction (μl)** | **Final concentration** |
| --- | --- | --- | --- |
| Nuclease-free water | Invitrogen (10320995) | 7 | N/A |
| Taq 2X master mix | NEB (M0270L) | 12.5 | 1X |
| 5S forward primer (20μM) | Eurofins | 1.25 | 1μM |
| 23S reverse primer (20μM) | Eurofins | 1.25 | 1μM |

Table S6 Cycling conditions optimised locally and based on the conditions described in the TaqMan Fast Universal PCR Mastermix Manual (#4366072, Applied Biosystems).

| Step Name | Temperature (°C) | Time (M:S) | Data Collection | Cycles |
| --- | --- | --- | --- | --- |
| Denature | 95 | 00:20 | None | 1 |
| Amplify | 95 | 00:03 | None | 40  40 |
|  | 60 | 00:30 | Hold Step |  |

Table S7 Primers and probes used for the detection of Borrelia spp., Coxiella burnetii, Francisella tularensis, Rickettsia spp., Tick-borne Encephalitis Virus.

| **Target organism** | **Target Gene/Region** | **Forward Primer (5'-3')** | **Reverse Primer (5'-3')** | **Probe (5'-3')** | **Reference** |
| --- | --- | --- | --- | --- | --- |
| Borrelia spp. | 16S rRNA | AGC CTT TAA AGC TTC GCT TGT AG | GCC TCC CGT AGG AGT CTG G | 6FAM-CCG GCC TGA GAG GGT GAA CGG-BHQ1 | [56] |
| Borrelia spp. | 5S-23S intergenic spacer | GAG TTC GCG GGA GAG TAG GTT ATT GCC | TCA GGG TAC TTA GAT GGT TCA CTT CC |  | [63] |
| Coxiella burnetii | COM1 | AAT CGC AAT ACG CTG CCA AA | AGC AGC GCG TCG TGG AA | 6FAM-AGC AGC CGC TAA ACA-BHQ1 | [57] |
| Coxiella burnetii | ICD | CGT TAT TTT ACG GGT GTG CCA | CAG AAT TTT CGC GGA AAA TCA | 6FAM-CAT ATT CAC CTT TTC AGG CGT TTT GAC CGG-BHQ1 | [58] |
| Coxiella burnetii | IS1111a | TAA CGG CGC TCT CGG TTT | TGC CGG GAA CGA TGA AA | 6FAM-TGA TGA ATG TCA CCC ACG CTC GCA-BHQ1 | [57] |
| Francisella tularensis | 23kDa | TGA GAT GAT AAC AAG ACA ACA GGT AAC A | GGA TGA GAT CCT ATA CAT GCA GTA GG | 6FAM-TCA GTT CTC ACA TGA ATG GTC TCG CCA-BHQ1 | [59] |
| Francisella tularensis | ISFtu2 | TTG GTA GAT CAG TTG GTG GGA TAA C | TGA GTT TTA CCT TCT GAC AAC AAT ATT TC | 6FAM-AAA ATC CAT GCT ATG ACT GAT GCT TTA GGT AAT CCA-BHQ1 | [59] |
| Francisella tularensis | Tul4 | ATT ACA ATG GCA GGC TCC AGA | TGC CCA AGT TTT ATC GTT CTT CT | TTC TAA GTG CCA TGA TAC AAG CTT CCC AAT TAC TAA G | [59] |
| Rickettsia spp. | gltA | TCG CAA ATG TTC ACG GTA CTT T | TCG TGC ATT TCT TTC CAT TGT G | 6FAM-TGC AAT AGC AAG AAC CGT AGG CTG GAT G-BHQ1 | [61] |
| TBEV | 3'non-coding region | GGG CGG TTC TTG TTC TCC | ACA CAT CAC CTC CTT GTC AGA CT | 6FAM-TGA GCC ACC ATC ACC CAG ACA CA-BHQ1 | [62] |

Table S8 Cycling conditions optimised locally and based on the conditions described by Drosten et al., 2002 [60]. PCR Kit: Superscript III Platinum One-Step qRT-PCR kit (#11745500, Invitrogen).

| Step Name | Analysis Mode | Temperature (°C) | Time (M:S) | Acquisition Mode | Cycles | Rate (°C/sec) |
| --- | --- | --- | --- | --- | --- | --- |
| Reverse Transcription | None | 45 | 10:00 | None | 1 | 20 |
| Denature | None | 95 | 05:00 | None | 1 | 20 |
| Amplify | Quantification | 95 | 00:05 | None | 45 | 20 |
|  | Quantification | 57 | 00:35 | Single | 45 | 20 |
| Cooling | None | 40 | 00:30 | None | 1 | 20 |
|  |  |  |  |  |  |  |

Table S9 Cycling conditions optimised locally and based on the conditions described in the Taq 2X Mastermix Manual (#M0270L, New England Biolabs).

| Step Name | Temperature (°C) | Time (M:S) | Cycles |
| --- | --- | --- | --- |
| Denature | 95 | 00:30 | 1 |
| Amplification I | 95 | 00:30 | 10  40  1 |
| Amplification II  Final Extension | 70  68  95  60  68  68 | 00:30  01:30  00:30  00:30  01:30  05:00 |  |

Table S10 Borrelia genotypes detected through the sequencing of the 5S-23S rRNA intergenic spacer.

| **Isolate** | **Genotype** | **GenBank Accession** |
| --- | --- | --- |
| IEPKUI-89-Hofn | *B. afzelii* | PV166197 |
| IEPKUI-289-Hofn | *B. garinii* | PV166198 |
| IEPKUI-301-Hofn | *B. garinii* | PV166199 |
| IEPKUI-302-Hofn | *B. garinii* | PV166200 |
| IEPKUI-329-Hofn | *B. garinii* | PV166201 |
| IEPKUI-376-Hofn | *B. garinii* | PV166202 |
| IEPKUI-429-Hofn | *B. garinii* | PV166203 |
| IEPKUI-492-Hofn | *B. garinii* | PV166204 |
| IEPKUI-503-Hofn | *B. garinii* | PV166205 |
| IEPKUI-533-Hofn | *B. garinii* | PV166206 |
| IEPKUI-536-Hofn | *B. garinii* | PV166207 |
| IEPKUI-542-Hofn | *B. garinii* | PV166208 |
| IEPKUI-660-Hofn | *B. garinii* | PV166209 |
| IEPKUI-108-Hofn | *B. garinii* | PV166210 |
| IEPKUI-109-Hofn | *B. garinii* | PV166211 |
| IEPKUI-110-Hofn | *B. garinii* | PV166212 |
| IEPKUI-112-Hofn | *B. garinii* | PV166213 |
| IEPKUI-121-Hofn | *B. valaisiana* | PV166214 |
| IEPKUI-122-Hofn | *B. garinii* | PV166215 |
| IEPKUI-123-Hofn | *B. garinii* | PV166216 |
| IEPKUI-127-Hofn | *B. garinii* | PV166217 |
| PHE-628-Hofn | *B. garinii* | PV166218 |
| PHE-650-Hofn | *B. garinii* | PV166219 |
| PHE-645-Hofn | *B. valaisiana* | PV175890 |
| IEPKUI-330-Hofn | *B. garinii* | PV190205 |
| IEPKUI-423-Hofn | *B. valaisiana* | PV190206 |
| IEPKUI-444-Hofn | *B. valaisiana* | PV190207 |
| IEPKUI-471-Hofn | B. garinii | PV190208 |
